# Editorial: New insights of cardiac rehabilitation: from basic to translational and clinical research

**DOI:** 10.3389/fcvm.2023.1217355

**Published:** 2023-06-02

**Authors:** Yingjie Zhang, Yu Zheng

**Affiliations:** Department of Rehabilitation Medicine, The First Affiliated Hospital of Nanjing Medical University, Nanjing, China

**Keywords:** cardiac rehabilitation, cardiovascular diseases, quality of life, CPET, exercise

**Editorial on the research topic** New insights of cardiac rehabilitation: from basic to translational and clinical research

Cardiac rehabilitation (CR) is a vital and established element of standard care in cardiology (1). It was first pioneered in the 1940s, facing opposition at some time (2). Efforts to shift the focus from bed rest to low-to-high intensity activities following a cardiac event marked the inception of what we currently recognize as CR. After emergency treatment for patients with cardiovascular diseases (CVDs), providing them with CR has been proven to improve clinical outcomes, prognosis, and quality of life (QoL) (3, 4). Nevertheless, results of CR remain controversial. Therefore, elaborating detailed mechanisms of CR on functional and structural remodeling after CVDs has important implications for routine application of CR.

This Research Topic entitled “New Insights of Cardiac Rehabilitation: From basic to translational and clinical research” covered a variety of aspects of CR for CVDs (from basic science, through clinical studies to treatment and intervention approaches).

The study by Gu et al. aimed to investigate the impact of anodal high-definition transcranial current stimulation (HD-tDCS) targeted at the left dorsolateral prefrontal cortex (DLPFC) on heart rate (HR) and heart rate variability (HRV) in healthy individuals. The participants were randomized to receive anodal HD-tDCS or sham HD-tDCS over the left DLPFC. A washout period of one week was incorporated to ensure validity of the results. The authors reported that anodal HD-tDCS applied over left DLPFC was capable to decrease HR and modulate HRV in healthy young individuals. These observations indicated that HD-tDCS showed promises in the regulation of cardiovascular function.

The purpose of study by Tan et al. was to explore the effect of blood flow–restricted resistance training on myocardial fibrosis in early spontaneously hypertensive rats (SHRs). The authors reported that in myocardium, blood flow–restricted low- and medium-resistance training (BFRT) was able to impede TGFβ-1-Smad pathway, downregulate CTGF expression, and regulate MMPs and TIMPs balance. These effects led to a reduction in myocardial fibrosis, improved cardiac morphology and function, and decreased blood pressure in SHRs, making it a potential effective approach for preventing and treating hypertension.

A previous study revealed that the expression of long non-coding RNA (lncRNA) LINC00452 was significantly reduced in blood samples of individuals with coronary artery disease (CAD). Nevertheless, the specific function of LINC00452 in the development of atherosclerosis (AS) requires further exploration. Yuan et al. reported that upregulation of LINC00452 could reverse oxLDL-induced growth suppression of HUVECs AS model through modulation of miR-194-5p/IGF1R axis. These results suggested that LINC00452 may serve as a promising therapeutic target for AS.

In the fourth study by Köteles et al. 55 patients with Metabolic Syndrome (MetS) were instructed to complete a 12-week home-based physical training with 3–5 sessions of exercise activity (min. 150 min) each week. Heart rate sensors, a fitness app, and a cloud-based data transfer system were used to remotely monitor the training sessions. The authors reported that waist and hip circumferences were decreased, exercise capacity increased, and mental health improved after intervention. They concluded that 12-week home-based physical training produced significant positive changes in several core components of individuals with MetS.

Yao et al. aimed to assess the usefulness of gray-scale ultrasound (US) and shear wave elastography (SWE) in assessing the condition of the skeletal muscles in patients with chronic heart failure (CHF). In this prospective study, quantitative US parameters of gastrocnemius medialis (GM) were compared using gray-scale US and SWE between CHF patients and healthy volunteers. Compared with the control group, there was a significant difference in fascicle length (FL), pinnation angle (PA), and echo intensity (EI) of the GM, but no statistically significant difference in Young's modulus values. Therefore, gray-scale US and SWE were expected to guide early rehabilitation training and improve patient prognosis.

The purpose of the protocol published by Zhang et al. was to assess the role of CR in patients with left ventricular aneurysm (LVA) formation after anterior MI. A single-center, single-blind, prospective, randomized controlled trial was proposed. A total of 36 sessions of CR will be delivered in the intervention group. The primary outcome was peak oxygen uptake measured by cardiopulmonary exercise testing. This study was expected to determine whether CR can improve physical capacity and clinical outcomes in patients with LVA formation after anterior MI. Another protocol proposed by Li et al. was a randomized controlled, open-label, single-centric prospective superiority trial which was aimed to assess the effects of an ultra-protective tidal volume on clinical outcomes compared to a protective tidal volume in patients receiving veno-arterial extracorporeal membrane oxygenation (VA-ECMO). This trial may provide an innovative mechanical ventilation strategy for VA-ECMO-supported patients.

Last but not the least, a review paper by Song et al. aimed to provide a comprehensive analysis of the cardiopulmonary exercise test (CPET). The authors reported that the number of published articles increased annually from 2002 to 2021, Americas and Europe led the field. It emphasized that heart failure, oxygen uptake, and prognostic value were the primary themes of these studies.

In conclusion, this Research Topic provides a relevant contribution in the field of CR to explore the therapeutic effects and potential mechanisms behind various CR interventions on cardiovascular diseases, laying the foundation for clinical application.
